# Sumoylation Inhibits the Growth Suppressive Properties of Ikaros

**DOI:** 10.1371/journal.pone.0157767

**Published:** 2016-06-17

**Authors:** Apostol Apostolov, Isma Litim-Mecheri, Attila Oravecz, Marie Goepp, Peggy Kirstetter, Patricia Marchal, Antoine Ittel, Laurent Mauvieux, Susan Chan, Philippe Kastner

**Affiliations:** 1 Institut de Génétique et de Biologie Moléculaire et Cellulaire (IGBMC), INSERM U964, CNRS UMR 7104, Université de Strasbourg, 67404 Illkirch, France; 2 Laboratoire d’Hématologie, Hôpitaux Universitaires de Strasbourg, Strasbourg, France; 3 Laboratoire d’Hématologie Cellulaire, EA 3430, Institut d’Hématologie et d’Immunologie, Faculté de Médecine de Strasbourg, Fédération de Médecine Translationnelle de Strasbourg, Université de Strasbourg, Strasbourg, France; 4 Faculté de Médecine, Université de Strasbourg, Strasbourg, France; Medical College of Wisconsin, UNITED STATES

## Abstract

The Ikaros transcription factor is a tumor suppressor that is also important for lymphocyte development. How post-translational modifications influence Ikaros function remains partially understood. We show that Ikaros undergoes sumoylation in developing T cells that correspond to mono-, bi- or poly-sumoylation by SUMO1 and/or SUMO2/3 on three lysine residues (K58, K240 and K425). Sumoylation occurs in the nucleus and requires DNA binding by Ikaros. Sumoylated Ikaros is less effective than unsumoylated forms at inhibiting the expansion of murine leukemic cells, and Ikaros sumoylation is abundant in human B-cell acute lymphoblastic leukemic cells, but not in healthy peripheral blood leukocytes. Our results suggest that sumoylation may be important in modulating the tumor suppressor function of Ikaros.

## Introduction

Sumoylation is a post-translational modification that involves the conjugation of small ubiquitin-like modifiers (SUMO1-3 in mammals) to target proteins. SUMO proteins function by modulating the activity and processes of target proteins, such as nuclear localization, transcriptional regulation and protein stability [1–3]. Indeed, sumoylation has been shown to modulate the function of transcription factors [4–6]. SUMO targets usually contain a consensus sumoylation ΨKxE/D motif, where Ψ is a hydrophobic amino acid [7].

The Ikaros zinc finger transcription factor is important for multiple aspects of hematopoiesis. Ikaros has been shown to act both as a transcriptional repressor and activator, by interacting with chromatin remodeling complexes like NuRD, PRC2 or SWI/SNF [8–10]. However, it remains largely unknown why Ikaros activates some genes and represses others. A potential mechanism may involve post-translational modifications. Indeed, phosphorylation has been shown to be important for Ikaros function in several systems [11–14]. Ikaros has also been reported to be sumoylated, and sumoylation has been proposed to prevent Ikaros from functioning as a repressor by preventing its association with transcriptional co-repressors [15].

Here we investigated the nature and function of Ikaros protein modifications in lymphocytes. We show that sumoylation is a major post-translational modification and identify three essential lysines in this process. Nuclear localization and DNA binding are required for sumoylation, and sumoylation reduces the ability of Ikaros to inhibit cell proliferation. Finally, we show that human leukemic cells exhibit high levels of sumoylated Ikaros.

## Materials and Methods

### Cell lines and primary cells

ILC87 cells [16] were maintained in RPMI1640 supplemented with 25 mM HEPES; 10% heat-inactivated fetal calf serum; 1 mM NaPyr; 1% Pen/Strep; 50μg/ml Gentamycin. The ILC87-derived cell lines were treated with 100 nM 4-hydroxytamoxifen (4OHT, Sigma) diluted in ethanol. ACC42, RS4;11 and Tom-1 cells [17–19] were cultured in RPMI1640 supplemented with 10% fetal calf serum and 50μg/ml Gentamycin. The primary B-ALL sample from an adult patient was cultured in presence of a monolayer of MS-5 stromal cells in MEM ALPHA 1900 supplemented with 10% fetal calf serum and Gentamycin. Written consent from the patient was obtained, and the study was approved by the Comité de Protection des Personnes "Est IV" (agreement # 09/20a). Retrovirus production and cell transduction was as described [20]. GFP-positive cells were sorted by FACS and further expanded. To avoid the skewing of the data by clonal selection, all experiments were performed with early passage cells (<15). ILC87-NGFR cells are mock-transduced with Mig-NGFR, which expresses an inert form of the human NGFR. Primary thymocyte populations were defined as DN3 (CD3-CD4-CD3-CD25+CD44-), DN4 (CD3-CD4-CD8-CD25-CD44-) and DP (CD4+CD8+), and purified by FACS using a Facs AriaII cell sorter (BD Biosciences).

### Microarray analysis

Total RNA was extracted with the RNeasy Micro kit (Qiagen), and 150 ng was used for transcriptome analysis on GeneChip^®^ Mouse Gene 1.0 ST arrays (Affymetrix) using standard procedures. Data were normalized with the Robust Multiarray Average algorithm. Probe sets that did not correspond to an identified gene were not included for analysis.

### Protein extract preparation

For total cell extracts, cells were harvested, washed once in ice cold PBS and lysed in RIPA buffer without DTT (50 mM Tris pH 8; 150 mM NaCl; 1% NP-40, 0.5% sodium deoxycholate, complete EDTA free protease inhibitor; Phosphatase inhibitor cocktail 3; 10 mM N-ethylmaleimide (NEM); 10 mM iodoacetic acid (IAA) (all reagents from Sigma)). Cytosolic extracts were prepared by incubating cells in hypotonic buffer (HEPES 10 mM, pH 7.9; 1.5 mM MgCl_2_; 10 mM KCl; complete EDTA free protease inhibitor; phosphatase inhibitor cocktail 3; 10 mM NEM; 10 mM IAA for 30 min on ice, vortexing each for 10 min. After spinning for 5 min at 13 000 rpm (4°C), the nuclear pellet was lysed in RIPA buffer to obtain the nuclear fraction. The lysate was then vortexed, spun for 10 min at 13 000 rpm (4°C), and the supernatant collected.

### Antibodies

Mouse monoclonal anti-estrogen receptor (ERa-F3) and rabbit polyclonal anti-Ikaros (C-terminal) antibodies were generated by the antibody facility of the IGBMC. Rabbit polyclonal anti-Ikaros (N-terminal) antibody (ab26083) was purchased from Abcam. Polyclonal rabbit anti-SUMO-1 and monoclonal rabbit anti-SUMO2/3 (18H8) antibodies were from Cell Signaling. Goat anti-mouse and goat anti-rabbit antibodies coupled to horseradish peroxidase were from Life Technologies.

### Western blotting

Protein extracts were mixed with 5x sample buffer to a final volume of 50 μl and loaded on SDS polyacrylamide gels (6, 8 or 10%) or NuPAGE Novex 3–8% Tris-Acetate gels (Invitrogen). Proteins were transferred to PVDF membranes (Immobilon P IPVH00010, Millipore), saturated with 5% non-fat milk for 1 hour at room temperature (RT) and probed overnight at 4°C with the indicated antibodies. Membranes were then washed three times (TBS; 0.1% Tween) and incubated with the appropriate secondary antibody in 5% non-fat milk for 1 hour at RT. After three washes, proteins were revealed using Western ECL Substrate (from Biorad or Pierce). To detect sumoylated proteins on membranes previously probed with anti-Ikaros or anti-ER antibodies, membranes were stripped in 62.5mM Tris pH 6.8, 100mM β-Mercaptoethanol, 2% SDS for 30 min at 50°C with agitation, washed three times, re-saturated and reprobed with anti-SUMO antibodies.

### Immunoprecipitation

500 μl of total protein extract prepared from 50x10^6^ cells were pre-cleared with 20 μl of 50% slurry protein A or protein G sepharose beads (Sigma) for 30 min at 4°C with rotation. Beads were pelleted by centrifugation for 1 min at 13 000 rpm (4°C) and extracts were transferred into tubes containing 100 μl of 50% slurry protein A or protein G sepharose beads and incubated overnight at 4°C with 5 μg of the indicated antibody. Beads were then spun (3 min, 2000 rpm, 4°C) and washed 5 x with RIPA lysis buffer (300 mM NaCl) without inhibitors. Beads were then boiled in 40 μl of 3x Laemmli sample buffer and loaded on the gel.

### Luciferase reporter assay

The luciferase reporter assay was performed as described previously [20].

## Results

### Ikaros proteins are modified in normal and leukemic T cells

**Fig 1:**
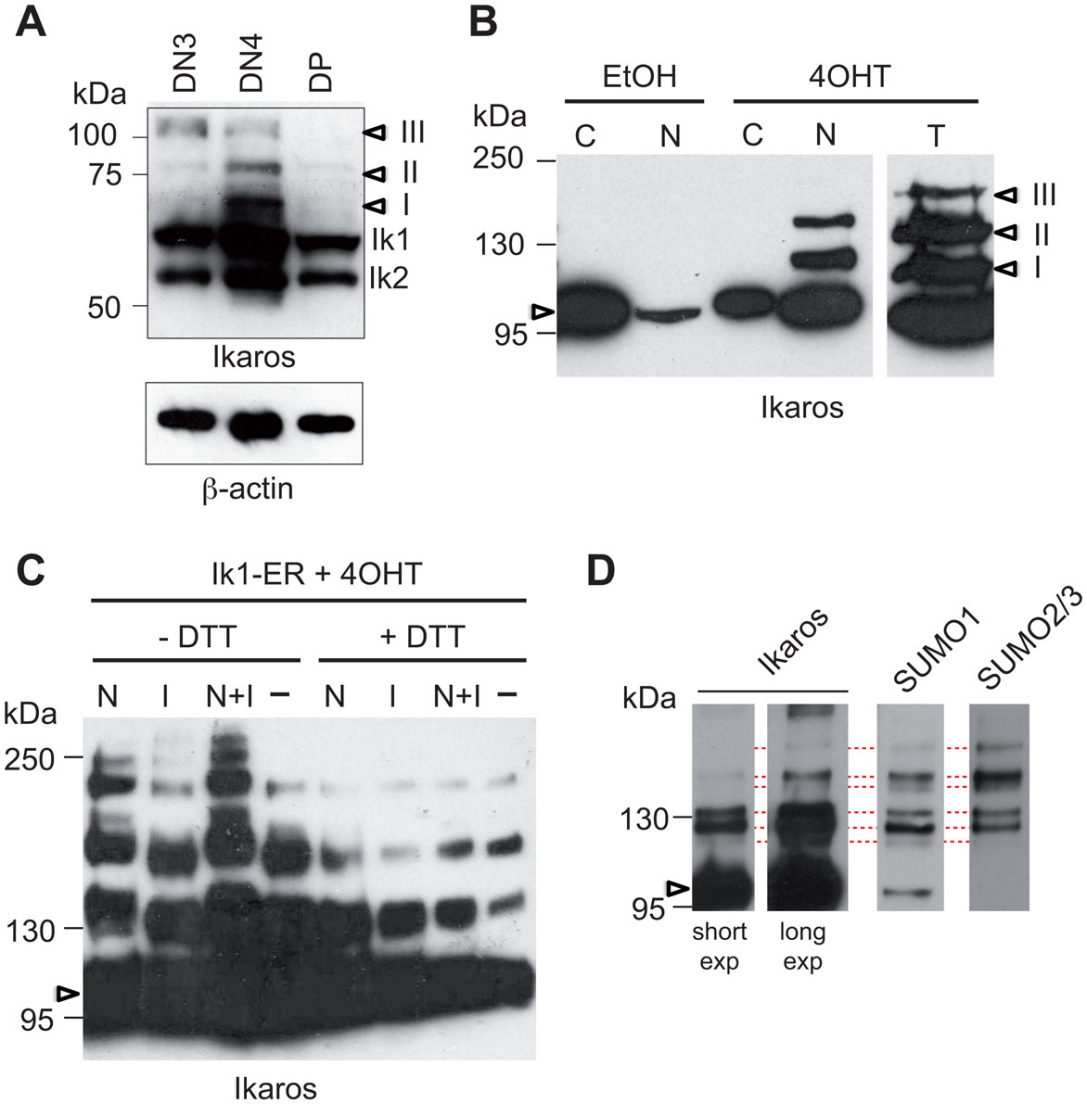
Ikaros is sumoylated in T cells. (**A**) 5x10^5^ DN3, DN4 or DP cells were lysed in 1x sample buffer, subjected to SDS-PAGE (8%), and probed with an anti-Ikaros antibody. Arrowheads point to the modified fractions. (**B**) Cytosolic (C), nuclear (N) or total (T) fractions from 10^6^ EtOH- or 4OHT-treated ILC87-IK1-ER cells were subjected to SDS-PAGE (10%) and analyzed with an anti-Ikaros antibody. (**C**) Western blot analysis (6% SDS-PAGE) with an anti-Ikaros antibody of total cell extracts from 2x10^6^ 4OHT-treated ILC87-IK1-ER cells that were lysed in the presence, absence or combination of N-ethylmaleimide (N), iodacetic acid (IAA, I) or 20 mM DTT, as indicated. (**D**) 4OHT-treated ILC87-Ik1-ER cells were lysed in buffer containing 1% NP-40 in the presence of NEM and IAA. Nuclear extracts from 50x10^6^ cells were immunoprecipitated with an anti-ER antibody and analyzed on a NuPAGE Novex 3–8% Tris-Acetate gel with the indicated antibodies. In (B), (C) and (D), arrowheads point to unmodified Ik1-ER proteins.

To study Ikaros modifications in developing T cells, we analyzed the relative size of these proteins in total extracts of CD4^-^CD8^-^CD25^+^CD44^-^ (DN3), CD4^-^CD8^-^CD25^-^CD44^-^ (DN4) and CD4^+^CD8^+^ (DP) thymocytes by Western blot (Fig 1a). Besides the Ikaros1 (Ik1) and Ikaros2 (Ik2) isoforms, three proteins of higher molecular (MW) weight (I-III) were detected. These latter bands were detected in all three thymocyte subsets but were most abundant in DN4 cells, suggesting that Ikaros is post-translationally modified in primary T cells.

To better understand the nature of these modifications, we studied the ILC87-Ik1-ER cell line, derived from an Ikaros null T cell tumor, and engineered to overexpress the full-length Ik1 isoform fused to the ligand-binding domain (LBD) of the estrogen receptor (Ik1-ER) and green fluorescent protein (GFP) [10]. Upon treatment with 4-hydroxytamoxifen (4OHT), an ER antagonist, for 24h, GFP^+^ cells exhibited enhanced nuclear translocation of Ik1-ER (Fig 1b), and little evidence of differentiation in terms of CD4 and CD8 expression (S1 Fig) [10]. As Ik1 expression also resulted in cell cycle exit [20, 21], thus recapitulating the described anti-proliferative effects associated with Ikaros, we performed biochemical analyses using this system. Interestingly, while extracts from 4OHT-treated ILC87-Ik1-ER cells showed the presence high molecular weight Ikaros proteins, those from vehicle-treated cells did not (Fig 1b), suggesting that Ikaros modifications occur in the nucleus.

Modified Ikaros proteins were labile, and required the presence of enzymatic inhibitors to increase their abundance in the extract preparations. We tested different inhibitors, and found that N-ethylmaleimide (NEM) and iodoacetic acid (IAA), which irreversibly modify thiol groups and inhibit the action of ubiquitin, Nedd8 and SUMO-specific isopeptidases [22–24], efficiently prevented the loss of the higher weight Ikaros proteins during protein preparation, compared with a single inhibitor or no treatment (Fig 1c). In contrast, DTT, which inhibits NEM and IAA activity, diminished the quantity of the modified proteins. Thus, protein extracts prepared in the presence of NEM and IAA, and in the absence of DTT, were studied in the experiments described below.

### Ikaros sumoylation requires DNA binding

Because Ik1 was previously reported to be sumoylated when overexpressed with SUMO1 in 293T cells [15], we looked for sumoylated Ikaros proteins in ILC87-Ik1-ER cells. Nuclear extracts from 4OHT-treated cells were immunoprecipitated (IP) with an anti-ER antibody, and then analyzed by Western blot with anti-Ikaros, anti-SUMO1 or anti-SUMO2,3 antibodies (Fig 1d). Both anti-SUMO antibodies recognized proteins of similar high MW size as the anti-Ikaros antibody, suggesting that Ikaros is sumoylated by SUMO1 and SUMO2,3 in leukemic T cells.

**Fig 2:**
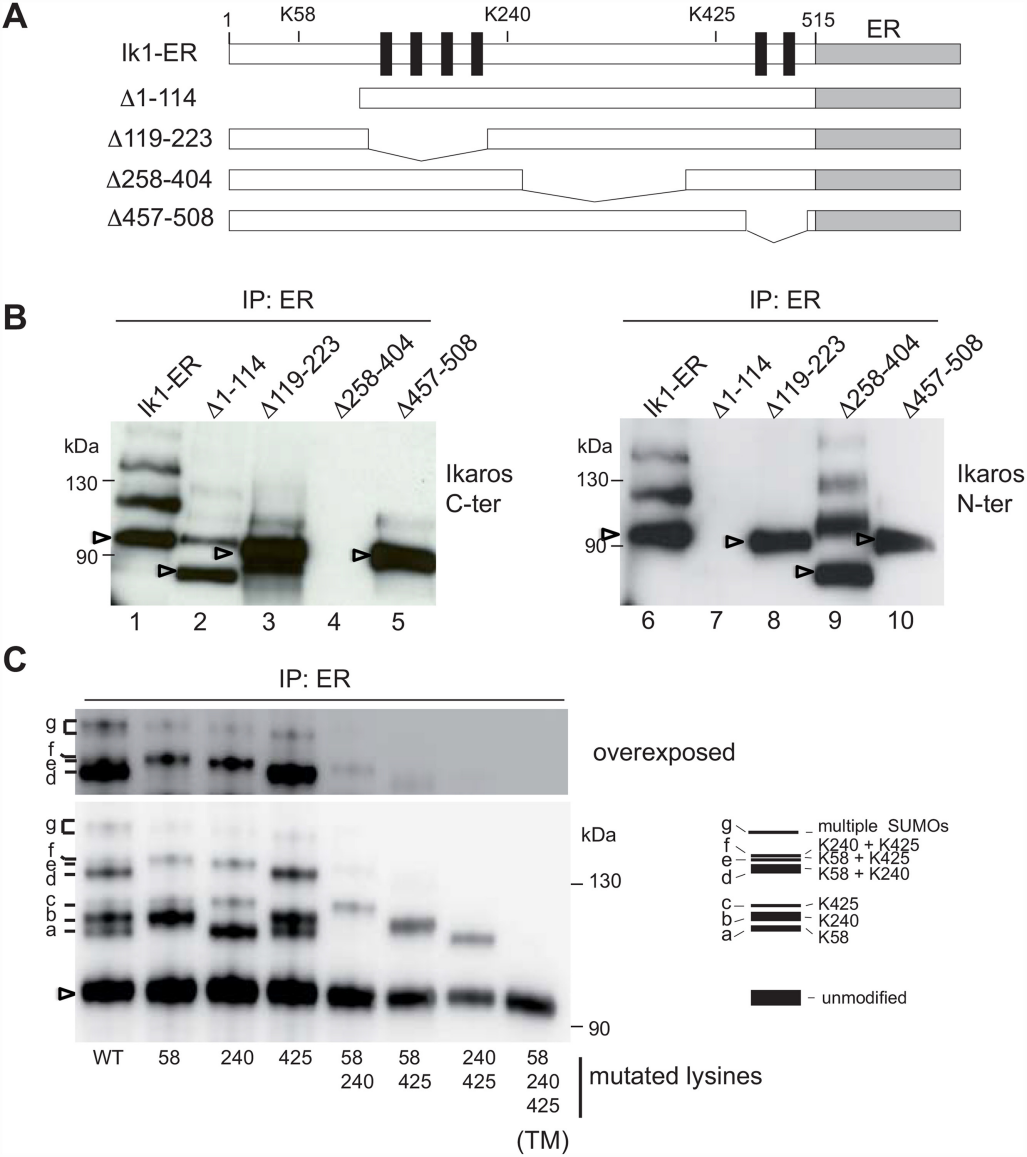
Impact of Ikaros mutations on sumoylation. **(A)** Schematic representation of Ik1-ER deletion mutants. (**B**) Modification pattern of Ik1-ER deletion mutants. ILC87 cells expressing Ik1-ER or deletion mutants were treated and lysed as described in Fig 1D. Nuclear extracts were immunoprecipitated with an anti-ER antibody, separated by SDS-PAGE (6%) and analyzed with anti-Ikaros antibodies against C-terminal or N-terminal epitopes. (**C**) Nuclear extracts from 50x10^6^ ILC87 cells expressing Ik1-ER or the indicated point mutants were immunoprecipitated with an anti-ER antibody, separated on a NuPAGE Novex 3–8% Tris-Acetate gel and analyzed with an anti-Ikaros antibody. The pattern of the various modified proteins is schematized on the right and the corresponding modifications indicated. In (B) and (C), arrowheads point to unmodified proteins.

To determine why nuclear localization is required for Ikaros sumoylation, we next investigated the influence of DNA binding and dimerization, by constructing Ik1-ER mutants lacking the N-terminal domain (aa 1–114), the DNA binding domain (DBD) (aa 119–223) [25], a large region between the DBD and the dimerization domain (aa 258–404), or the dimerization domain (aa 457–508) (Fig 2a). Although the ability of Ikaros to localize to the nucleus was unaffected by these mutations (S2 Fig), we found that loss of the DBD or the dimerization domain prevented the appearance of modified Ikaros (Fig 2b; compare lanes 3 and 5 with lane 1, or lanes 8 and 10 with lane 6). In contrast, the deletion of aa 258–404 did not affect the levels of the high MW Ikaros proteins (compare lane 9 with lane 6), while deletion of ww 1–114 reduced, but did not abolish, their presence (compare lane 2 with lane 1). These results indicated that the sumoylation of Ikaros is linked to it's ability to bind target gene sequences.

### Sumoylation occurs on lysines 58, 240 and 425

Our bioinformatic analyses predicted that the Ikaros protein sequence contains three ΨKxE/D consensus sumoylation sites at lysines 58, 240 and 425. K58 and K240 were previously found to be targets of sumoylation [15]. We therefore mutated all 3 lysines to arginines (K→R), individually or in combination, and expressed the resulting proteins fused to the ER LBD in ILC87 cells. These cell lines were treated with 4OHT, and the nuclear extracts were immunoprecipitated with the anti-ER antibody and analyzed for Ikaros, SUMO1 and SUMO2,3 (Fig 2c and S3 Fig). These experiments showed that the anti-Ikaros, anti-SUMO1 and anti-SUMO2,3 antibodies recognized similar bands in the different extracts, as expected (S3 Fig). Of the seven detectable bands (labeled a-g) in the Ik1-ER extracts, mutation of K58 led to the loss of bands a, d and e, mutation of K240 led to the loss of bands b, d and f, and mutation of K425 led to the loss of bands c, e and f (Fig 2c). This suggested that bands a, b and c corresponded to single sumoylation events at K58, K240 and K425, respectively, while bands d, e and f were the result of bi-sumoylation. These results were confirmed with extracts from cells expressing double mutant Ik1 proteins where only one of the lysines was left untouched. The triple mutation (TM) of K58, K240 and K425 led to the loss of all higher MW Ikaros proteins, including band g which was detected in cells expressing single K mutants, but not in those expressing the double or triple mutants (see overexposed blot in Fig 2c). This band may correspond to a mixture of Ikaros proteins with multiple SUMO moities on 2 or 3 of the lysine residues. Importantly, the absence of modified proteins in cells expressing the TM-ER indicated that the ER end of the fusion protein is not a significant target of sumoylation. Thus, K58, K240 and K425 all contribute to Ikaros sumoylation.

### Sumoylation of Ikaros has little effect on gene expression

**Fig 3:**
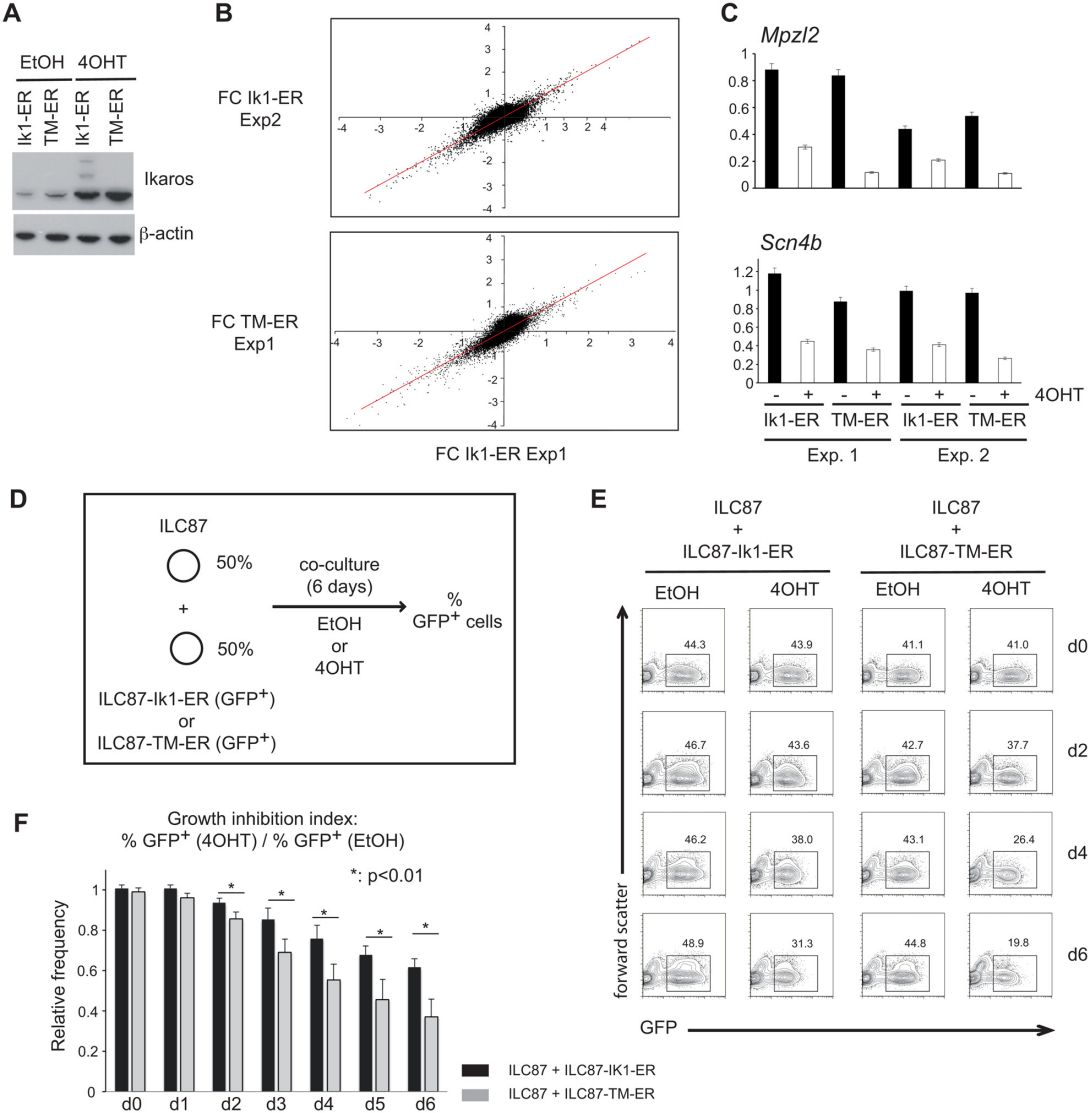
Sumoylation limits the growth-inhibitory effects of Ikaros. **(A)** Western blot showing similar level of Ik1-ER and Ik-TM-ER proteins in nuclear extracts of ILC87-Ik1-ER and ILC87-Ik-TM-ER cells treated with EtOH or 4OHT. **(B)** Scatter plots showing the distributions of the fold changes (4OHT- vs EtOH-treated cells, expressed as log2 values) in the 2 independent analyses performed with ILC87-Ik1-ER cells (top panel), or between representative analyses performed with ILC87-Ik1-ER and ILC87-Ik-TM-ER cells (bottom panel). The red diagonal highlights the theoritical position for probe sets with similar fold-changes. **(C)** RT-qPCR analysis of repression of the *Mpzl2* and *Scn4b* genes in ILC87-Ik1-ER and ILC87-Ik-TM-ER cells treated with 4OHT for 24h, in 2 independent experiments (duplicate measurements in each case). **(D-F)** Competitive growth inhibition assay. **(D)** Experimental setup: ILC87 cells transduced with IK1-ER or Ik-TM-ER (GFP^+^) were mixed at a 1:1 ratio with ILC87 cells (or ILC87 cells mock-transduced with an empty Mig-NGFR retrovirus) and cultured for 6 days in the presence of EtOH or 4OHT. **(E)** Proportion of GFP^+^ cells in living cells of EtOH and 4OHT-treated ILC87-Ik1-ER and ILC87-Ik-TM-ER cells in a representative experiment. **(F)** Growth inhibition over time by Ik1-ER and Ik-TM-ER (ratio of GFP^+^ cells in 4OHT-treated over EtOH-treated samples; average of 4 experiments). Statistical significance was evaluated with a Student's t-test.

To begin to understand the functional consequences of sumoylation on Ikaros, we first investigated its effects on the ability of Ikaros to regulate gene expression. ILC87-Ik1-ER or ILC87-Ik-TM-ER cells were purified to express similar levels of GFP and Ikaros (Fig 3a), then treated or not with 4OHT, and their transcriptomes were analyzed in two independent experiments (Fig 3b). In ILC87-Ik1-ER cells, 89 genes were upregulated and 237 were downregulated after 4OHT treatment (fold-change >2 in each experiment). Unexpectedly, similar results were obtained with 4OHT-treated ILC87-Ik-TM-ER cells. A few genes appeared to be changed in the ILC87-Ik-TM-ER cells, but these differences were not reproduced in a third independent transcriptome experiment (not shown). Our results thus suggested that sumoylation does not significantly affect the way Ikaros regulates gene expression at the genome-wide level in ILC87 cells.

At the single gene level, we analyzed the mRNA levels of *Mpzl2* and *Scn4b*, two genes shown to be repressed by Ikaros [10], in ILC87-Ik1-ER and ILC87-Ik-TM-ER cells. No detectable effect of sumoylation was observed by RT-qPCR, as the expression of these genes was also similarly downregulated in both cell lines after 4OHT treatment (Fig 3c). Finally, we evaluated the ability of the TM protein to repress the activity of the *Hes1* promoter following Notch pathway activation in transient luciferase reporter transfection experiments, as had been shown for Ik1 [20]. HeLa cells were transfected with a Hes1-luciferase reporter plamid, and expression vectors encoding the intracellular domain of Notch1 (ICN1), and Ik1 or TM (S4 Fig). These results indicated that loss of sumoylation had little detectable impact on the ability of Ikaros to repress the transcription of the Hes1-luciferase reporter.

Altogether, our results indicate that sumoylation does not play a major role in the ability of Ikaros to modulate gene expression in this system.

### Sumoylated Ikaros is less effective at inhibiting cell growth

We then evaluated the consequences of Ikaros sumoylation on leukemic cell expansion. As Ik1 re-expression in Ikaros null leukemic cells inhibits their expansion [20, 21] (S5 Fig), we compared the capacity of TM-ER and Ik1-ER to inhibit leukemic cell expansion in competitive co-culture assays. ILC87-Ik1-ER or ILC87-Ik-TM-ER cells with similar GFP levels were co-cultured with Ikaros null ILC87 cells (GFP^-^) at a 1:1 starting ratio over a 6 day period, and the proportions of GFP^+^ cells were analyzed in the presence or absence of 4OHT (Fig 3d and 3e). In vehicle-treated cultures, the ratio of GFP^-^ and GFP^+^ cells remained steady over time (Fig 3e). However, in 4OHT-treated cultures, the proportion of GFP^+^ cells decreased more in ILC87-Ik-TM-ER than ILC87-Ik1-ER co-cultures. This difference was confirmed when we calculated the growth inhibition (GI) index between the percentage of GFP^+^ cells in the 4OHT-treated and vehicle-treated cultures (Fig 3f, S1 Table), which showed that the GI index of Ik-TM-expressing cells was higher than that of Ik1-expressing cells. These results indicated that unsumoylated Ikaros inhibits cell growth better than sumoylated Ikaros.

### Human B-ALL cells show high levels of sumoylated Ikaros

**Fig 4:**
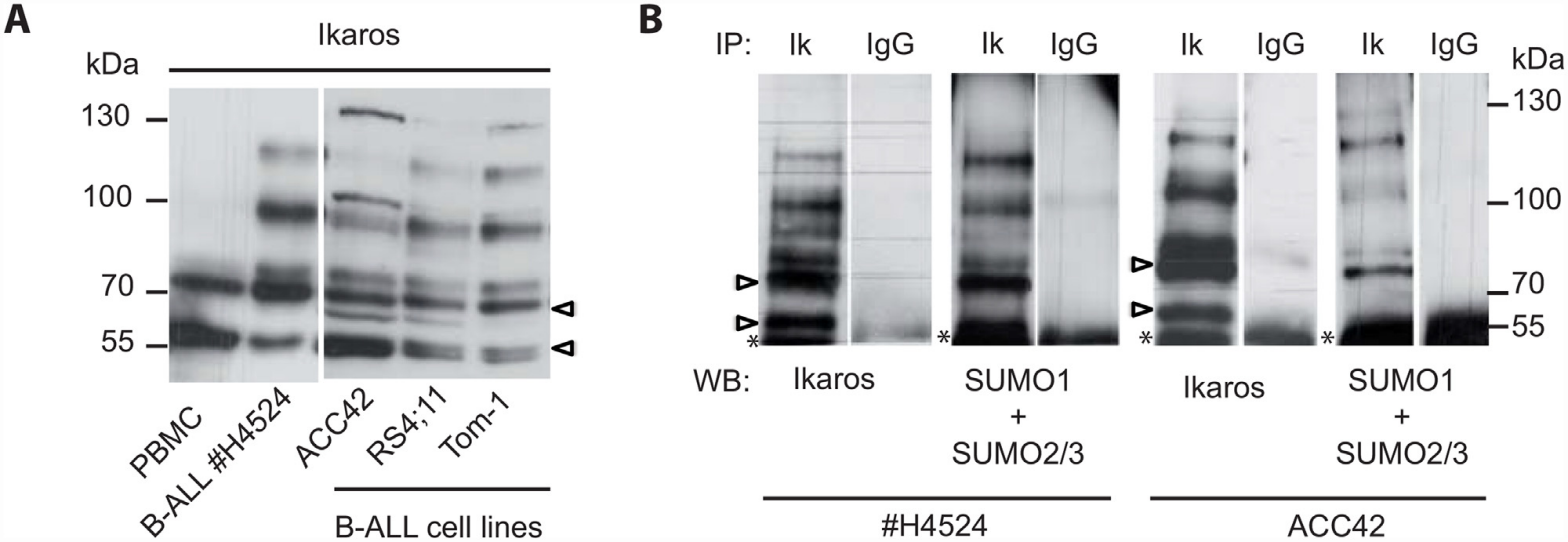
Sumoylation of Ikaros in B-ALL cells. **(A)** Detection of Ikaros proteins by western blot in whole cell extracts from human peripheral blood mononuclear cells (PBMC), primary leukemic cells from a B-ALL patient, and the ACC42, RS4;11 and Tom-1 B-ALL cell lines. **(B)** Detection of Ikaros sumoylation in whole cell extracts from B-ALL patient #H4524 and the cell line ACC42. Protein extracts were immunoprecipitated with an anti-Ikaros antibody, and probed with an anti-Ikaros antibody, or with a mix of anti-SUMO1 and anti-SUMO2/3 antibodies. Open arrowheads point to the Ik1 and Ik2 isoforms; asterisks to IgGs. Note that a sumoylated protein comigrates with Ik1, presumably corresponding to sumoylated Ik2.

The above experiments suggested that sumoylation reduces the ability of Ikaros to inhibit cell proliferation. We therefore evaluated the sumoylation state of Ikaros in human B-ALL cells, where loss of Ikaros function is a major event [26]. Total cell extracts from three B-ALL cell lines and one primary B-ALL sample were analyzed with an anti-Ikaros antibody by Western blot, and compared with peripheral blood mononuclear cells from 2 healthy donors (Fig 4a and not shown). The B-ALL samples used possessed either wild-type *IKZF1* alleles (patient #4524, ACC42) or monoallelic *IKZF1* deletions that resulted in haploinsufficiency (Tom-1, RS4;11), as determined by multiplex ligation-dependent probe amplification (not shown). Interestingly, Ikaros was abundantly modified in B-ALL cells, but not in PBMCs. These modified Ikaros proteins were sumoylated because they were also detected with antibodies to SUMO1 and SUMO2,3, in extracts immunoprecipitated first with an anti-Ikaros antibody (Fig 4b). The complex pattern of sumoylated bands may reflect sumoylation of both the Ik1 and Ik2 isoforms (S6 Fig). These results showed that B-ALL cells contain high levels of sumoylated Ikaros, regardless of whether their *IKZF1* alleles are mutated, suggesting an important role for Ikaros sumoylation in leukemic cell development.

## Discussion

Sumoylation is a major post-translational modification that influences protein activity, and we provide evidence that sumoylation may negatively affect the tumor suppressor function of Ikaros through, in part, a decrease in cell growth inhibition. Ikaros is markedly sumoylated in human B-ALL cells, and sumoylation occurs at three conserved lysine residues that act as SUMO1 and/or SUMO2,3 acceptor sites. Further, sumoylation requires nuclear localization and most likely DNA binding.

Our results confirm and extend previous findings in embryonic kidney and osteosarcoma cell lines that identified K58 and K240 as SUMO acceptor sites on the Ikaros protein [15]. Interestingly, K425 was not sumoylated in these cells, perhaps because the GFP-SUMO1 protein transfected into these cells preferentially sumoylates K58 and K240 due to its larger size (38 vs. 11kD for endogenous SUMO1). Alternatively, K425 sumoylation may require cell type-specific co-factors, as has been shown for the glucocorticoid receptor [27].

Sumoylation of Ikaros requires the DNA binding and dimerization domains. Since the three sumoylated lysines lie outside these domains, our results suggest that Ikaros sumoylation may be linked to DNA binding. The requirement for DNA binding in sumoylation has been observed for the yeast GCN4 protein, where sumoylation occurs after DNA binding to promote GCN4 clearance from promoter regions [28]. The concept that sumoylation may act to reduce transcription at specific gene subsets was also recently observed in human fibroblasts where sumoylation was inhibited [29]. Whether sumoylation affects Ikaros similarly remains to be investigated.

Ikaros sumoylation was previously associated with a loss of repressive properties, as Ikaros proteins conjugated with GFP-SUMO1 interacted poorly with co-repressors [15]. We were therefore surprised to find that cells expressing sumoylated Ikaros and those with unsumoylated Ikaros exhibit similar gene expression profiles by different criteria (transcriptome, RT-qPCR, luciferase assays), suggesting that sumoylation has minimal impact on gene activation or repression by Ikaros, and may be more modulatory than absolute in its influence. Alternatively, the transcriptional effects of sumoylation might be masked be the heterogeneity of the cell population that we studied, in particular with respect to clonal diversity and cell cycle status. It is for instance possible that sumoylation may play a role only at a certain stage of the cell cycle. This may be especially relevant as other studies have also revealed an inhibitory role of sumoylation in the transcriptional regulation of cell proliferation [29, 30]. Future studies will therefore be required to better understand the molecular importance of sumoylation on Ikaros in the regulation of genes associated with cell proliferation.

Nonetheless, our results indicate that sumoylation limits certain physiological functions associated with Ikaros. As Ikaros is abundantly sumoylated in B-ALL cells, it is thus tempting to speculate that leukemic cells might hijack this mechanism to reduce Ikaros activity during cancer development.

## Supporting Information

S1 Fig Analysis of the differentiation of ILC87-Ik1-ER cells after treatment with 4OHT for 24h. Surface CD4 and CD8 expression of ILC87-Ik1-ER cells treated with EtOH or 4OHT for 24h. Staining of cells from a WT thymus is shown as a positive control. Numbers in the respective gates are percentages.
(EPS)

S2 Fig Nuclear translocation of mutant Ik1-ER protein. Cytoplasmic and nuclear extracts of the indicated cells lines were prepared after treatment with EtOH or 4OHT (72h), and analyzed by western blotting with antibodies against N-terminal or C-terminal Ikaros epitopes. B-actin is shown as a loading control for each blot. Note that nuclear translocation is less efficient for the Δ119–223 mutant that lacks the DBD.
(EPS)

S3 Fig Sumoylation of lysine mutant Ik1-ER proteins by SUMO1 and SUMO2/3. Similar experiment as the one shown in Fig 2C. Extracts immunoprecipitated with an anti-ER antibody were separated on 2 separate gels that were probed with anti-SUMO1 and anti-SUMO2/3. The membrane probed with the anti-SUMO1 antibody was then stripped and to be probed with the anti-Ikaros antibody. Blue, green and red arrows point to proteins with mono-sumoylations on K58, K240 and K425, respectively.
(EPS)

S4 Fig Inhibition of Hes1 promoter activity by Ikaros1 and the TM mutant. HeLa cells were transfected with the indicated constructs and analyzed for luciferase activity. Results correspond to two independent experiments performed in triplicates.
(EPS)

S5 Fig 4OHT-dependent inhibition of ILC87-Ik1-ER cell growth. Cummulative cell numbers of ILC87-Ik1-ER and ILC87 cells cultured for 4 days in the presence of EtOH or 4OHT. Data are the mean+/- SD of 3 independent experiments.
(EPS)

S6 Fig Identification of human Ik1 and Ik2 isoforms. Ikaros proteins from whole cell extracts from human PBMC and B-ALL sample #H4524 were analyzed by western blot next to control Ik1 and Ik2 proteins produced by transfection of the corresponding expression vectors into Cos-1 cells.
(EPS)

S1 Table Growth inhibition by Ik1-ER and TM-ER. The Table provides the percentage of GFP-positive and negative cells at day 6 in 4 competition experiments between ILC87-Ik1-ER or ILC87-TM-Ik1-ER cells and empty ILC87 cells or mock-transduced ILC87-NGFR cells (see Fig 3c for experimental setup). Values in the "growth inhibition" columns correspond to the ratio of the percentages of GFP^+^ cells in 4OHT- over those in EtOH-treated samples.
(DOCX)
