# Correction: Sumoylation Inhibits the Growth Suppressive Properties of Ikaros

**DOI:** 10.1371/journal.pone.0160134

**Published:** 2016-07-21

**Authors:** The PLOS ONE Staff

The ninth author, Susan Chan, should also be listed as a corresponding author with the following email address: scpk@igbmc.fr. The publisher apologizes for the error.
