# Supplementary figures and images for: Sumoylation Inhibits the Growth Suppressive Properties of Ikaros
Source: PLoS One. 2016 Jun 17;11(6):e0157767. doi: 10.1371/journal.pone.0157767 (PMC4912065; doi:10.1371/journal.pone.0157767)

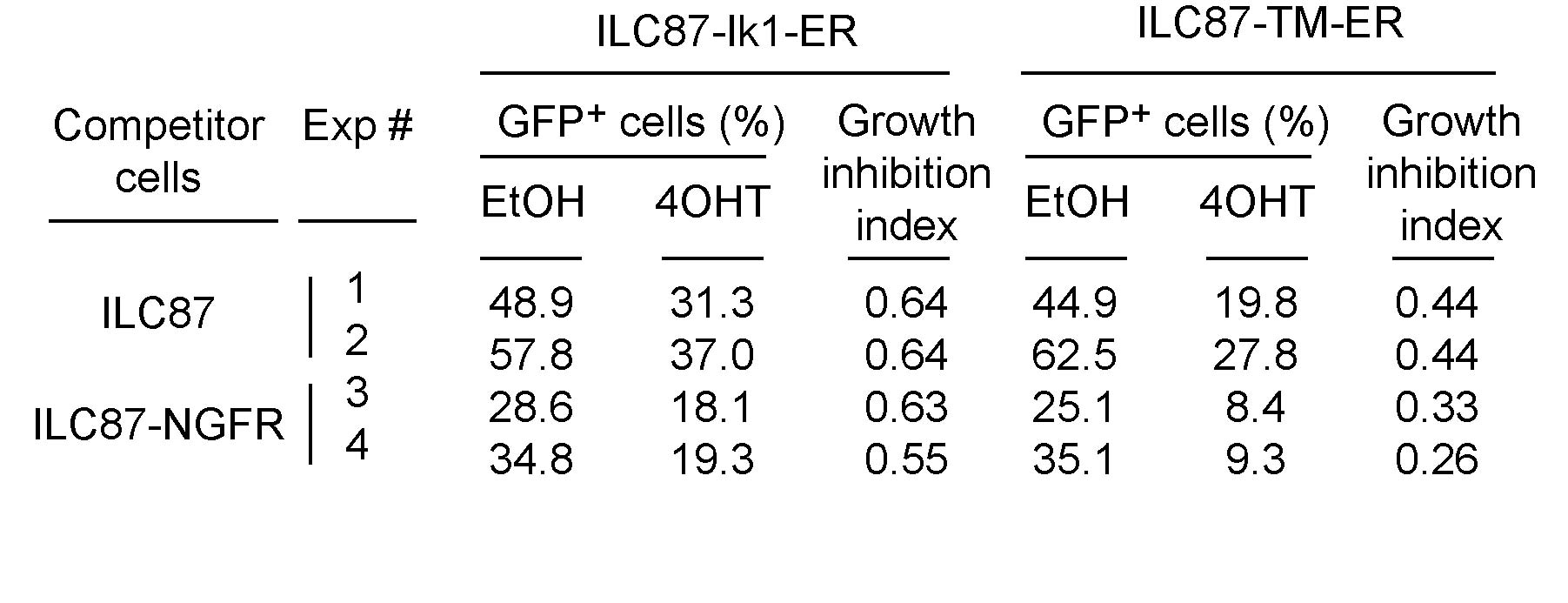

Supplement: S1 Table — The Table provides the percentage of GFP-positive and negative cells at day 6 in 4 competition experiments between ILC87-Ik1-ER or ILC87-TM-Ik1-ER cells and empty ILC87 cells or mock-transduced ILC87-NGFR cells (see Fig 3c for experimental setup). Values in the "growth inhibition" columns correspond to the ratio of the percentages of GFP+ cells in 4OHT- over those in EtOH-treated samples. (DOCX) [file pone.0157767.s007.docx]
